# Electrolyte Effects on N‑Heterocyclic Imines as the Redox Sorbent for Electrochemical CO_2_ Capture

**DOI:** 10.1021/acscentsci.6c00765

**Published:** 2026-09-03

**Authors:** Fang-Yu Kuo, Gi Hyun Byun, Betar M. Gallant

**Affiliations:** † Department of Chemical Engineering, 2167 Massachusetts Institute of Technology, Cambridge, Massachusetts 02139, United States; ‡ Department of Mechanical Engineering, Massachusetts Institute of Technology, Cambridge, Massachusetts 02139, United States

## Abstract

N-Heterocyclic imines (NHIs) represent a promising class of sorbents for electrochemically mediated carbon capture (EMCC) due to their ability to bind CO_2_ in the neutral state and operate reversibly at redox potentials more positive than that of oxygen. In this study, the effect of electrolyte composition on the thermochemical and electrochemical behavior of NHI-based systems is examined. Using reaction calorimetry and Raman and NMR spectroscopy, we demonstrate that Lewis acid cations stabilize the NHI–CO_2_ adduct through charged interactions, and the solvent and anion donor number tune this stabilization by modulating the effective cation Lewis acidity. As a result, electrolyte salts can substantially increase the magnitude of the NHI–CO_2_ reaction enthalpy, leading to higher CO_2_ loading on NHI at low CO_2_ partial pressure (10^–3^ atm) and faster capture kinetics. The impacts of the electrolyte selection on NHI redox behavior as well as oxygen reduction were also investigated. The findings indicate that electrolyte design must balance the strength of cation–NHI–CO_2_ stabilization, NHI redox activity, and the redox potential separation from oxygen reduction. By providing a mechanistic framework for how individual electrolyte components govern important process parameters, this work offers insights for optimizing electrolyte environments to achieve high-efficiency and reversible EMCC.

## Graphical abstract

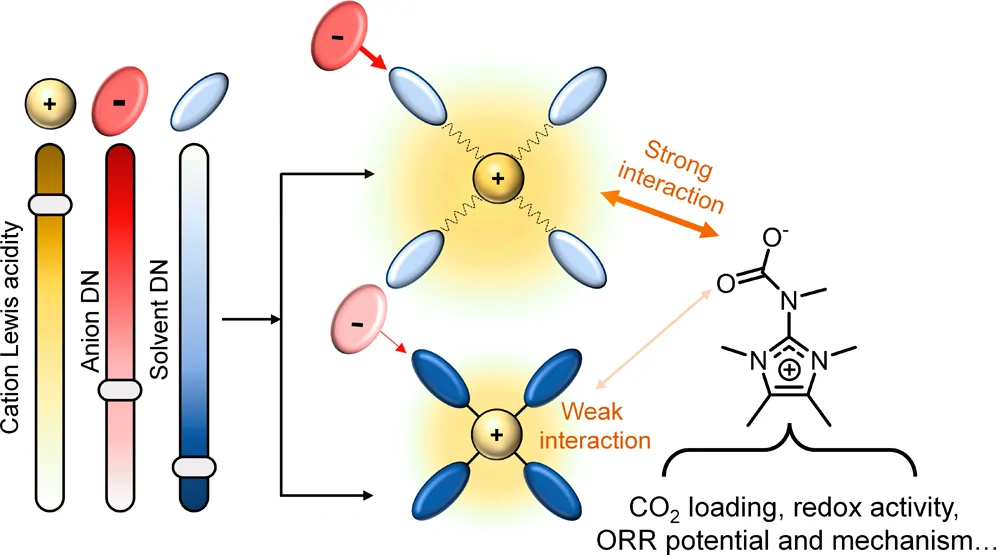

## Introduction

Electrochemically mediated carbon capture (EMCC) has gained increased attention as an emerging approach for CO_2_ separation that circumvents the thermal regeneration requirements of conventional amine scrubbing.^1,2^ In EMCC systems, electrical potentials are used to reversibly alter the chemical state of capture agents, enabling CO_2_ uptake and release under ambient temperature and pressure conditions while using electricity as the primary energy input. Operationally, the system works by circulating a liquid sorbent between absorption/desorption contactors—where CO_2_ is chemically bound/released—and an electrochemical cell, where targeted oxidation or reduction triggers CO_2_ desorption and regenerates the active species. A widely explored class of EMCC platforms employs redox-active organic molecules dissolved in nonaqueous electrolytes.^3,4^ These sorbents (e.g., quinone,^5^ azopyridine,^6^ isoindigo^7^) become strongly nucleophilic and bind CO_2_ after electroreduction, while oxidation restores the neutral sorbent and liberates the captured gas. Despite this concept’s proven demonstration, most reported organic sorbents operate at substantially negative reduction potentials (−1.25 to −2.0 V vs Fc/Fc^+^), placing them in direct competition with oxygen reduction (−1.25 V vs Fc/Fc^+^) and leading to parasitic side reactions that erode efficiency and durability.^8^

EMCC performance is governed not only by the intrinsic properties of the sorbent but also by the electrolyte environment where capture/release occurs. Desirable electrolytes should provide sufficient ionic conductivity, maintain the solubility of the sorbent and its charged intermediates, offer a wide electrochemical stability window, and minimize parasitic reactions. Beyond this general consideration, nuanced interactions between the sorbent and electrolyte components can also affect capture and regeneration behavior, making sorbent–electrolyte codesign an important strategy for improving EMCC systems. The importance of this approach is demonstrated by the following example.

In our previous work, we reported a distinct capture-oxidation EMCC platform using N-heterocyclic imines (NHIs) as redox-active sorbents.^9^ In contrast to conventional reductive-activation schemes, NHI directly binds CO_2_ in the neutral state and undergoes oxidation to trigger the CO_2_ release. This inversion of the electrochemical and chemical steps was found to substantially shift the operative redox potential to a more positive regime (e.g., −0.6 V vs Fc/Fc^+^), alleviating the need for strongly reducing potentials. In the same study, it was observed that the CO_2_ binding behavior of redox-reversible NHI can be highly sensitive to the electrolyte environment. Specifically, pairing a strong Lewis acidic cation, Li^+^, with weakly coordinating solvents such as acetonitrile (MeCN) enabled tuning of CO_2_ capacity on bis­(NHI) (phenylene-linked bis­(N-heterocyclic imine)) from negligible loading to as high as 2 mol CO_2_/mol NHI under 100% CO_2_ conditions.

Electrolyte effects on CO_2_ capture thermodynamics have been similarly observed for other classes of EMCC sorbents. Prior studies have primarily relied on empirical screening of electrolyte salts—typically within a single solvent—to shift the reduction potential of EMCC sorbents to more positive values, as determined by monitoring changes in cyclic voltammetry.^5,7,10,11^ In general, however, the concerted effect of cation, anion and solvent selection on the sorbent behavior has not been systematically studied for EMCC systems broadly nor for NHI systems specifically.

In this work, we quantitatively examine how electrolyte components influence the reaction enthalpy between NHI and CO_2_ and develop mechanistic interpretations linking these thermodynamic changes to specific solvation and ion-pairing interactions. We further connect these fundamental thermodynamic trends to performance considerations, including the CO_2_ loading capacity and redox behavior of NHI. Finally, we evaluate how electrolyte composition affects oxygen reduction behavior, a key consideration in EMCC sorbent design that has not received sufficient attention, despite its central role in determining operational stability. Together, these results establish a new understanding of electrolyte engineering considerations for optimizing capture strength, regeneration, and oxygen tolerance in future EMCC systems using NHI redox sorbents.

## Results and Discussion

In this study, an NHI derivative featuring *tert*-butyl side chains and an isopropyl group at the exocyclic position was selected as a model compound (Figure 1) for which the reaction enthalpy of CO_2_ binding (Δ*H*_NHI–CO2_) in salt-free MeCN was previously determined to be −84 kJ/mol CO_2_.^12^ Among NHI structures studied so far, the previously studied bis­(NHI) exhibited optimal performance characteristics considering multiple metrics like redox potential (500 mV more positive than oxygen reduction), excellent redox reversibility, and modulation of up to 2 CO_2_ per electron, however,this particular NHI structure only binds CO_2_ in Li^+^/MeCN electrolyte. The limited design space therefore precludes a systematic electrolyte study, which is why we focus here on a representative though globally nonoptimized structure to develop the necessary fundamental understanding. Nevertheless, because these compounds share a common CO_2_ binding motif at the exocyclic nitrogen, insights gained from the selected NHI are expected to be readily transferable to future redox-reversible NHI derivatives, particularly as molecular modifications on NHI expand feasible NHI-electrolyte combinations.

**Figure 1:**
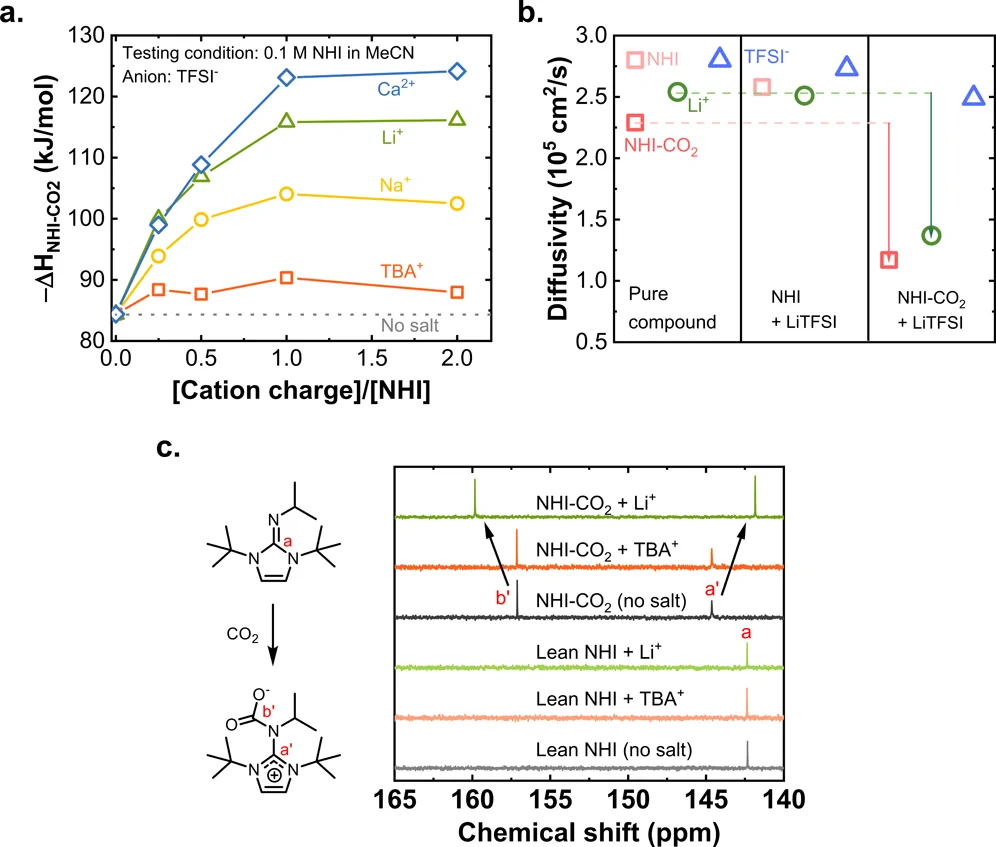
(a) Effect of cation identity and concentration on the reaction enthalpy between NHI and CO_2_ (Δ*H*_NHI–CO2_) at 100% CO_2_, measured with a micro reaction calorimeter using electrolytes containing 0.1 M NHI and 0.1 M ATFSI (A = TBA^+^, Na^+^, Li^+^) or Ca­(TFSI)_2_ in MeCN. (b) Diffusivity of electrolyte components (NHI, NHI–CO_2_, Li^+^, and TFSI^–^), determined by DOSY NMR for individual species (pure compound) and NHI/Li^+^ mixtures under Ar and CO_2_. (c) ^13^C NMR spectra of lean NHI and CO_2_-loaded NHI (0.1 M) in salt-free MeCN and in the presence of 0.1 M TBA^+^ and Li^+^ salts.

We first examine the effect of cation identity on the reaction enthalpy between NHI and CO_2_ (Δ*H*_NHI–CO2_) using MeCN and TFSI^–^ as the initial choice of the solvent and counteranion. In these experiments, the total concentration of NHI was fixed at 0.1 M, while the cation concentration was increased gradually to a [cation charge]/[NHI] ratio of 2, corresponding to 0.2 M total cation charge (i.e., 0.2 M salt for monovalent cations and 0.1 M for divalent cations). As shown in Figure 1a, Δ*H*_NHI–CO2_ is negative and the magnitude (exothermicity) increases proportionally with increasing cation concentration in all cases. Meanwhile, the magnitude of the enhancement scales with the Lewis acidity of the cation in the order TBA^+^ < Na^+^ < Li^+^ < Ca^2+^. For Li^+^ and Ca^2+^,^13^ the magnitude of Δ*H*_NHI–CO2_ (graphed as −Δ*H*_NHI–CO2_ for simplicity) increases from approximately 85 kJ/mol CO_2_ to 115–125 kJ/mol CO_2_, corresponding to a substantial stabilization of 30–40 kJ/mol CO_2_. We note that fixed [cation charge]/[NHI] comparisons do not strictly maintain constant ionic strength, so the effect of activity coefficients and electrostatic screening may influence the absolute Δ*H*_NHI–CO2_ values, especially for Ca^2+^. Nevertheless, the clear trend among monovalent cations under comparable formal ionic strength and the stronger Ca^2+^ effect are most consistent with increasing Lewis acid cation–carbamate stabilization as the dominant contributor given that Ca^2+^ has lower activity than the monovalent cations, which would otherwise predict lower interaction enthalpy. This modulation, achieved by salt modification only, is remarkable as it is comparable to the enthalpy changes attainable by modification of the sorbent structure itself, not only for NHI but also for other classes of sorbents (e.g., amines).^14,15^ Interestingly, our observation that strong Lewis acid cations stabilize CO_2_ binding contrasts sharply with trends reported for reductively activated sorbents (e.g., quinones and sp^2^ Lewis bases).^6,11^ In those systems, strong Lewis acid cations form tight ion pairs with the reduced sorbent directly, blocking CO_2_ binding sites and limiting practical capture performance. Although Ca^2+^ more strongly stabilizes the NHI–CO_2_ adduct, Li^+^ was selected for the following studies because Li-based electrolytes are widely studied in nonaqueous electrochemical systems and are commercially available with a broad range of high-purity anions, enabling reliance on well-established characterization methods and systematic anion comparison.

To elucidate the origin of the large enthalpy stabilization of NHI–CO_2_ with Lewis acid cations present, we first probe intermolecular interactions in the system by examining the diffusivities of NHI and Li^+^ using DOSY NMR, as shown in Figure 1b (anion effects will be discussed later).^16^ In MeCN, the diffusivity of NHI, NHI–CO_2_, and Li^+^ were individually measured as 2.80, 2.29, and 2.58 × 10^–5^ cm^2^ s^–1^, respectively, providing baseline values for the unassociated species. Under an Ar atmosphere, the diffusivities of NHI and Li^+^ in mixed NHI and Li^+^ electrolyte showed only a slight decrease upon mixing, indicating that direct interactions between NHI and Li^+^ are minor in the absence of CO_2_. In contrast, under a CO_2_ atmosphere, the diffusivity of NHI–CO_2_ and Li^+^ both decrease substantially to similar values of 1.17 and 1.37 × 10^–5^ cm^2^ s^–1^, respectively. This pronounced and correlated decrease in diffusivity indicates that there is a majority population of Li^+^ and NHI–CO_2_ that diffuses as an associated species on the NMR time scale, reflecting a strong charge interaction that is consistent with salt-stabilized association of the NHI–CO_2_ adduct.^17,18^ ^7^Li NMR experiments further support the strong charge interaction between Li^+^ and NHI–CO_2_ adducts (Figure S1), as evidenced by the apparent downfield shift and peak broadening of the Li resonance upon addition of NHI–CO_2_ to LiTFSI/MeCN electrolyte. The downfield shift is consistent with the association between Li^+^ and the NHI–CO_2_ adduct, and the peak broadening also suggests a more heterogeneous or asymmetrical local environment around Li^+^ and/or fast exchange between bulk-solvated and adduct-associated Li^+^ species.^19,20^

^13^C NMR spectroscopy was further employed to probe changes in the local electronic environment of NHI and NHI–CO_2_ upon introduction of Lewis acidic cations (Figure 1c). TBA^+^ and Li^+^ are used as the examples, and spectra for all other cations can be found in Figure S2. Under an Ar atmosphere, the addition of either a weak (TBA^+^) or a strong (Li^+^) Lewis acid does not change the chemical shift of NHI, as evidenced by the same resonance of carbon **a** in both cases. This observation indicates that direct NHI–cation coordination is negligible in the absence of CO_2_. Upon exposure to CO_2_, a new resonance (carbon **b′**) appears, corresponding to the CO_2_-derived carbamate carbon in the NHI–CO_2_ adduct. In this CO_2_-bound state, the presence of TBA^+^ maintains the chemical shifts of either **a′** or **b′** relative to the no-salt condition. In contrast, the introduction of Li^+^ induces a strong downfield shift of **b′** and a smaller upfield shift of **a′**. The significant Li^+^-specific downfield shift from 157 to 160 ppm of the carbamate carbon **b′** provides direct evidence for inner-sphere coordination of NHI–CO_2_ adduct to the Li^+^, in which Li^+^ interacts directly with the carbamate oxygen atoms and withdraws electron density from the adjacent carbon center.^21^ Upon Li^+^ coordination to the carbamate oxygen atoms, the anionic carbamate moiety is stabilized, reducing charge separation within the zwitterionic NHI–CO_2_ adduct between the carbamate anion and the cationic NHI core. This charge redistribution effectively increases the electron density of carbon **a′** located at the NHI core, leading to its upfield shift. Together with the DOSY results, these data establish that the NHI–CO_2_ adduct enters the first solvation shell of Li^+^.

The effect of anion identity on the thermodynamics of NHI–CO_2_ binding was next examined using either TBA^+^ or Li^+^ salts paired with various anions (PF_6_^–^, BF_4_^–^, ClO_4_^–^, TFSI^–^, and OTf^–^) in the NHI solution. As shown in Figure 2a, Δ*H*_NHI–CO2_ exhibited a strong dependence on the anion selection when Li^+^ was employed as the countercation. Δ*H*_NHI–CO2_ became increasingly exothermic from −109 kJ/mol (LiPF_6_) to −118 kJ/mol (LiOTf), which is positively correlated with the anion donor number (DN) from −6.2 (PF_6_^–^) to 20.4 kcal/mol (OTf^–^).^22^ Conversely, Δ*H*_NHI–CO2_ remained nearly constant when using the TBA^+^ cation, ranging between −89 and −91 kJ/mol. This cation-sensitive behavior suggests that a direct electrostatic interaction between the anion and the positively charged NHI core is likely absent, as such an interaction would provide a consistent anion effect regardless of the cation used. The absence of strong anion–NHI–CO_2_ interactions is also supported by the negligible change in TFSI^–^ diffusivity in the NHI–CO_2_ + Li^+^/MeCN electrolyte relative to pure MeCN (Figure 1b) and additional spectroscopy data (following).

**Figure 2:**
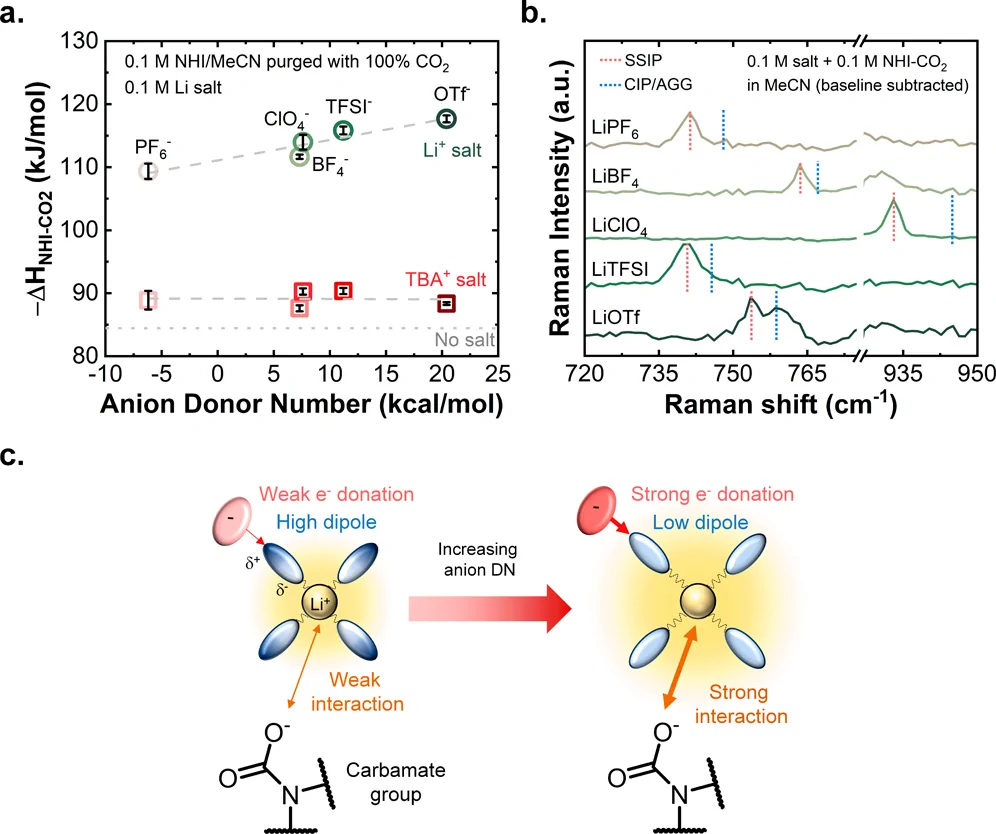
(a) Dependence of −Δ*H*_NHI–CO2_ on anion donor number. Samples containing 0.1 M NHI in 0.1 M TBAX/MeCN or LiX/MeCN (X = PF_6_^–^, BF_4_^–^, ClO_4_^–^, TFSI^–^, and OTf^–^) were purged with 100% CO_2_, and the heat release was measured using a micro reaction calorimeter. The data are presented as mean values ± standard deviation from three independent measurements. (b) Raman shift of 0.1 M NHI–CO_2_ in 0.1 M LiX/MeCN (X = PF_6_^–^, BF_4_^–^, ClO_4_^–^, TFSI^–^, and OTf^–^). The spectral window focuses on the anion vibrational modes, identifying solvent-separated ion pairs (SSIPs) and contact ion pairs/aggregates (CIPs/AGGs). All traces are corrected to an MeCN background. (c) Schematic illustration of how increasing anion donor number modifies the solvent polarity, leading to change of the solvation environment.

Raman spectroscopy was employed to provide further insight into the solvation environment in the electrolyte (Figure 2b).^23–25^ With the exception of the OTf^–^ system (Figure S3), all anions were found to exist as solvent-separated ion pairs (SSIPs).^23–25^ This indicates that Li^+^ remains fully coordinated by MeCN in its first solvation shell while the anions reside in the secondary shell, which confirms the absence of direct Li^+^–anion contact. The absence of direct NHI–anion or Li^+^–anion interactions suggests that the observed anion effect is instead mediated through the modulation of solvent–cation coordination. We propose a molecular mechanism for this phenomenon in Figure 2c based on an inductive effect of the solvent. In the primary shell, the solvent molecules develop an induced dipole to screen the Li^+^ charge.^26^ While low-DN anions exert minimal influence on this screening, high-DN anions act as potent Lewis bases, donating electron density to the MeCN molecules in the first solvation shell. This electronic induction attenuates the solvent’s induced dipole moment toward the Li^+^ center, effectively withdrawing the electron density that would otherwise screen the cation. Consequently, the Li^+^ ion retains a higher effective positive charge, which enhances its electrostatic stabilization of the NHI–CO_2_ adduct.

Having established the individual roles of cations and anions, we then fixed the salt to be LiTFSI and examined the effect of solvent choice. The solubility of both NHI and the NHI–CO_2_ adduct across a range of solvents was examined first. NHI–CO_2_ is found to be poorly soluble in low–dielectric constant solvents,^27,28^ such as triglyme and diethyl carbonate (Figure S4); consequently, subsequent measurements are conducted in solvents that provide at least 0.1 M solubility for NHI–CO_2_. The solvents that are examined include MeCN, propylene carbonate (PC), γ-butyrolactone (GBL), dimethylformamide (DMF), and dimethyl sulfoxide (DMSO). As shown in Figure 3a, in the absence of added salt, the measured Δ*H*_NHI–CO2_ exhibits negligible variation across different solvents (ranging from −83 to −84 kJ/mol CO_2_), indicating that solvent identity alone does not influence CO_2_ binding to NHI. On the other hand, upon addition of LiTFSI, the magnitude of Δ*H*_NHI–CO2_ progressively decreases from −115 to −86 kJ/mol as the solvent donor number (DN) increases (Figure 3a).^27,28^ As discussed above, a larger enthalpy magnitude in low DN solvents (e.g., −115 kJ/mol CO_2_ in 0.1 M LiTFSI/MeCN) originates from strong electrostatic interactions between Li^+^ and the carbamate anion formed upon CO_2_ binding. However, as the solvent DN increases, the extent of cation solvation by solvent molecules is enhanced.^29^ This strong solvation effect, as illustrated schematically in Figure 3b, substantially reduces the effective Lewis acidity of the cation and its interaction with the carbamate group, thereby weakening the stabilization effect in the high DN solvents. Notably, the salt-induced change in the magnitude of Δ*H*_NHI–CO2_ does not change linearly with solvent donor number as with anion DN but, instead, drops most sharply in the DN ≈ 14–20 kcal/mol regime between MeCN, PC, and GBL. This steep drop is followed by a more gradual leveling-off at higher DN: e.g., −87 and −86 kJ/mol CO_2_ for DMF and DMSO, respectively. This is presumably because, once Li^+^ is sufficiently strongly screened by solvent, subsequent screening has a negligible effect.

**Figure 3:**
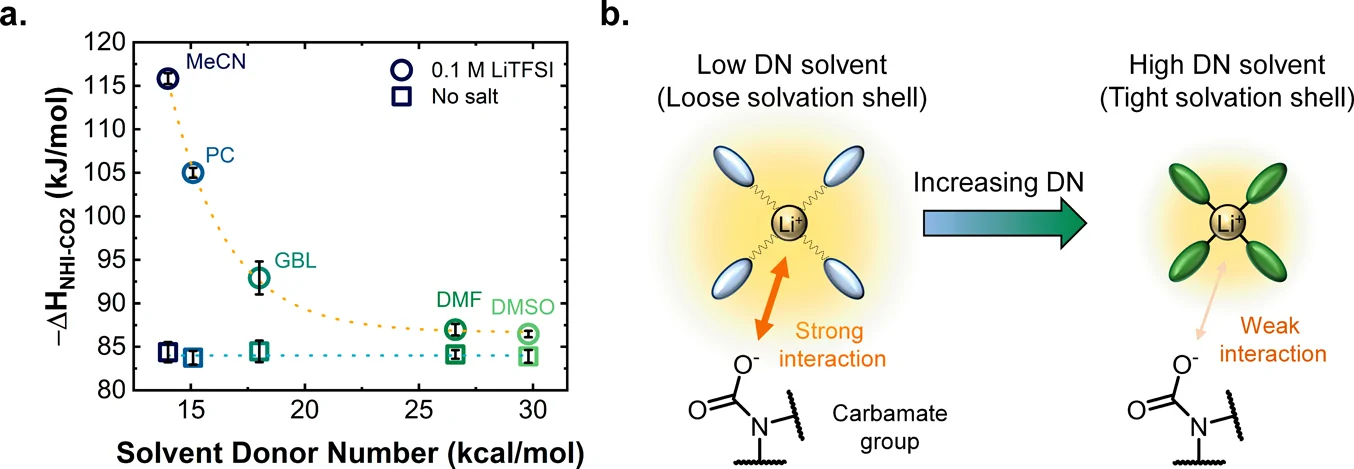
(a) Influence of solvent donor number on −Δ*H*_NHI–CO2_ in the absence and presence of 0.1 M LiTFSI in MeCN-based electrolytes. All samples contain 0.1 M NHI and are purged with 100% CO_2_ to measure the heat release using the micro reaction calorimeter. Data is presented as mean values ± standard deviation from three independent measurements. (b) Schematic illustration of the impact of low vs high solvent donor number on the cation solvation environment, which in turn strengthens or attenuates cation–NHI–CO_2_ interactions, respectively.

Having established the effect of individual electrolyte components on Δ*H*_NHI–CO2_, we next examined how the increase in the Δ*H*_NHI–CO2_ magnitude can benefit practical applications. To start, we measure the achievable CO_2_ loading of NHI with and without 0.1 M LiTFSI in PC. PC is selected instead of MeCN due to its high boiling point, which minimizes solvent loss and sensor interruption during CO_2_ loading measurements (experimental setup in Figure S5), and the CO_2_ loading of NHI was measured under 10^–3^–10^–2^ atm CO_2_ partial pressure. In the absence of salt, we obtained CO_2_ loadings of 0.54, 0.74, 0.88, and 0.93 mol CO_2_/mol NHI, while in the presence of the salt, these loadings increase to 0.76, 0.90, 0.96, and 0.99 mol CO_2_/mol NHI (Figure 4). Raw data can be found in Figures S6–S7. These increases are substantial, especially at 10^–3^ atm CO_2_ partial pressure, where we achieve an additional 0.22 mol CO_2_/mol NHI. Note that the improvement gradually shrinks under 5 × 10^–3^–10^–2^ atm partial pressure, primarily because the NHI approaches full loading of CO_2_ in these conditions. We fitted the CO_2_ loadings over various CO_2_ concentrations to obtain the equilibrium constant *K* and further calculate the Gibbs free energy of the NHI CO_2_ capture reaction (see Supporting Information for definition of *K* and fitting methods). We found only a modest change in the Gibbs free energy magnitude from −17.8 kJ/mol to −19.9 kJ/mol NHI upon comparing solutions without and with salt, respectively, as shown in Figure 4, which is much more closely spaced than the differences in enthalpy found in Figures 1–3. The apparent insensitivity of Gibbs free energy to the added cation indicates that the large enthalpic stabilization that arises from salt incorporation is largely offset by an unfavorable entropy loss. This entropy penalty is consistent with strong cation–carbamate interactions that reduced the configurational entropy in the bound state.^30,31^ Nevertheless, the difference in Gibbs free energy without and with salt (∼3 kJ/mol) is still sufficient to cause notable differences in *K* (1.3 ± 0.3 × 10^3^ and 3.6 ± 1.1 × 10^3^, respectively) and CO_2_ loading as discussed.

**Figure 4:**
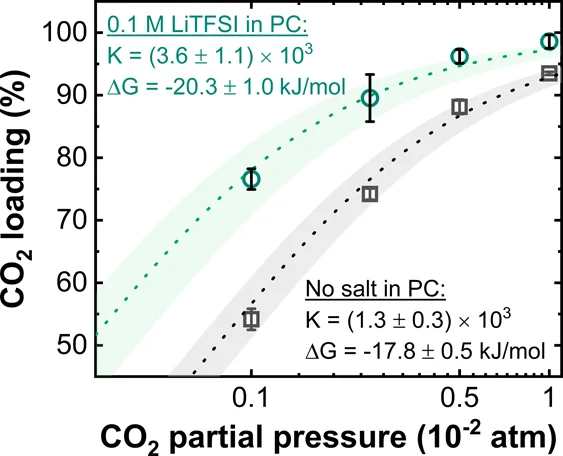
CO_2_ loading of 0.1 M NHI in the absence and presence of 0.1 M LiTFSI in PC electrolytes under 10^–3^–10^–2^ atm CO_2_ partial pressure. Shaded region represents 95% confidence interval for the fit.

The effect of electrolyte composition on the electrochemical characteristics of NHI and NHI–CO_2_ adducts was investigated by cyclic voltammetry (CV) in electrolytes with varying solvent DN: high (DMSO) and low (PC and MeCN) and 0.1 M salt (TBATFSI or LiTFSI). CV measurements provide insight into the redox potential of NHI–CO_2_ during electrochemical cycling for CO_2_ capture/release and electron-transfer kinetics subject to differing extents of NHI–CO_2_–cation interaction strength. Note that the NHI used for the aforementioned electrolyte screening suffers from electrochemical irreversibility due to a hydrogen-abstraction reaction following NHI oxidation, which prevents subsequent reduction.^9^ Therefore, for CV measurements, an alternative NHI derivative with moderate CO_2_ binding strength (Δ*H*_NHI–CO2_ = −57 kJ/mol CO_2_ in the absence of salt) was used to ensure sufficient electrochemical reversibility for accurate determination of both reduction and oxidation behaviors (Figure 5; NHI structure in inset).^12^

**Figure 5:**
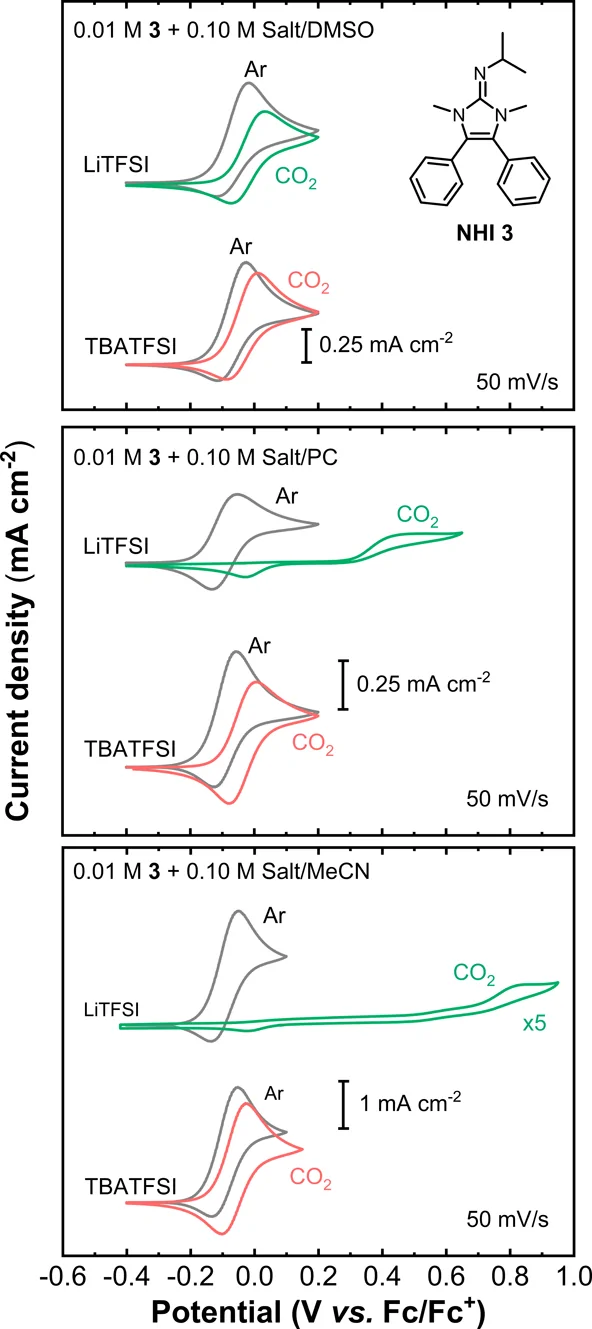
Cyclic voltammograms of 0.01 M NHI recorded in electrolytes containing 0.1 M LiTFSI or TBATFSI in DMSO, PC, and MeCN under Ar and CO_2_ atmospheres at a scan rate of 50 mV s^–1^. Working electrode: glassy carbon; counter electrode: Pt; reference electrode: Ag/Ag^+^. The chemical structure of NHI **3** is shown in the inset at the top.

Under Ar (gray traces), CV curves collected in the same solvent show invariant redox potential and peak currents across TBA^+^ and Li^+^, which is consistent with there being minimal interaction between the cation and the lean NHI in the absence of CO_2_, as discussed in Figure 1. The typical peak-to-peak splitting is 80–90 mV, indicating acceptable reversibility for meaningful study of electrolyte effects. In contrast to performance under Ar, under CO_2_, drastically different electrochemical behaviors are observed between TBA^+^ and Li^+^ electrolytes. In all solvents with TBA^+^ as the cation, CO_2_ binding causes a slight positive shift (30–60 mV) in the oxidation potential and a 10–25% decrease in the peak current. We attribute these changes to a minor decrease in electron density at the exocyclic nitrogen and the diffusion coefficient, respectively, upon CO_2_ binding, compared to the lean NHI.

However, when Li^+^ is used as the cation, the magnitude of the potential shift with CO_2_ depends strongly on solvent donor number. In low-DN solvents (PC and MeCN), a significant positive shift (∼0.5–0.85 V) in oxidation potential is observed, along with a decrease in oxidation current and wider peak separation (∼0.4–0.8 V). This behavior reflects the combined effects of thermodynamic stabilization of the NHI–CO_2_ adduct and hindered electron-transfer kinetics, both arising from strong Li^+^–NHI–CO_2_ interactions.^32^ By comparison, in high-DN DMSO, changes in both oxidation potential and current are minimal compared to the experiments conducted under Ar, which is attributed to the strong solvation of Li^+^ by DMSO. Overall, these results highlight a practical consideration for tuning the electrolyte environment of NHI-based EMCC. Although strong Lewis acidic cations in low-DN solvents significantly enhance the magnitude of Δ*H*_NHI–CO2_ and CO_2_ loading, they can impose a kinetic barrier and energetic penalty in the EMCC regeneration process by overstabilizing the bound state. Furthermore, the increase in anodic–cathodic peak separation resulting from salt stabilization will also increase the energy penalty of the CO_2_ separation process. Therefore, we envision careful balancing will be required when using salt stabilization as a strategy to tune the enthalpy of NHI–CO_2_ adduct formation in future electrolyte design.

While the above results clearly demonstrate the impact of the supporting electrolyte (salt and solvent) selection on EMCC behavior, prior work in the field of lithium–oxygen batteries has extensively demonstrated that changing the electrolyte composition can markedly influence the oxygen reduction reaction (ORR, −1.00 to −1.50 vs Fc/Fc^+^) in nonaqueous media.^33,34^ This point has, to the best of our knowledge, received apparently little emphasis in the EMCC literature so far. We therefore examined (1) how the presence of the NHI redox sorbent affects the ORR in various electrolyte environments;^35–37^ and (2) given that O_2_ and NHI redox potentials are unlikely to shift identically for a given cation, whether there exist circumstances where the ORR and NHI reduction reaction can become insufficiently separated, enabling competition or even parasitic reactions between the two once O_2_ becomes activated. We note that the ORR potential is often reported as being around −1.20 to −1.30 V vs Fc/Fc^+^ in nonaqueous EMCC systems;^6,7^ though subject to the above considerations, it is emphasized that this is only approximate.^38,39^

Oxygen reduction was examined in two solvents, DMSO and ACN, representative of a high and low DN solvent, respectively. In DMSO, ORR has been previously found to proceed via a solution-mediated mechanism in which O_2_ is reduced to superoxide (O_2_^–^) and becomes quasi-stably solvated by the solvent in the electrolyte (Figure 6a).^35,36^ In TBA^+^ electrolytes, the superoxide can persist over hour-long time scales.^40,41^ In contrast, in alkali cation-based electrolytes, a disproportionation reaction occurs in the bulk solution that leads to more-rapid production of peroxide (A_2_O_2_, A = alkali metal–cation) and concurrent O_2_ release, corresponding to 2 e^–^/O_2_ overall.^35,36^ Crucially, the electrochemical potential in DMSO is governed by the initial O_2_/O_2_^–^ redox and is thus cation-independent (potentials of −1.25 to −1.30 V vs Fc/Fc^+^), which is attributable to the high coordinating tendency of DMSO toward alkali cations, rendering their reactivity to be as comparably low as TBA^+^ toward the production of peroxide.^33,41^ In contrast, in low DN solvent such as MeCN, the relatively lower reduction potential of the ORR with TBA^+^ cations (−1.50 V vs Fc/Fc^+^) is shifted positively when using Na^+^ or Li^+^ (∼−1.30 V vs Fc/Fc^+^), demonstrating a stronger cation effect.^33,41^ ORR in low-DN solvents occurs via a surface-mediated pathway in which reduced oxygen species form surface-adsorbed NaO_2_ or LiO_2_ due to insufficient solvation toward the cation.^33,35^ This is followed by further electrochemical reduction to Na_2_O_2_ or Li_2_O_2_ (Figure 6a), which forms as a solid on the working electrode that can lead to electronic passivation and corresponding to an identical 2 e^–^/O_2_ as with the solution-mediated reaction.^38,39^ This second electron transfer registers the nature of the cationic species stabilizing the superoxide, leading to cation-dependent reduction potentials.

**Figure 6:**
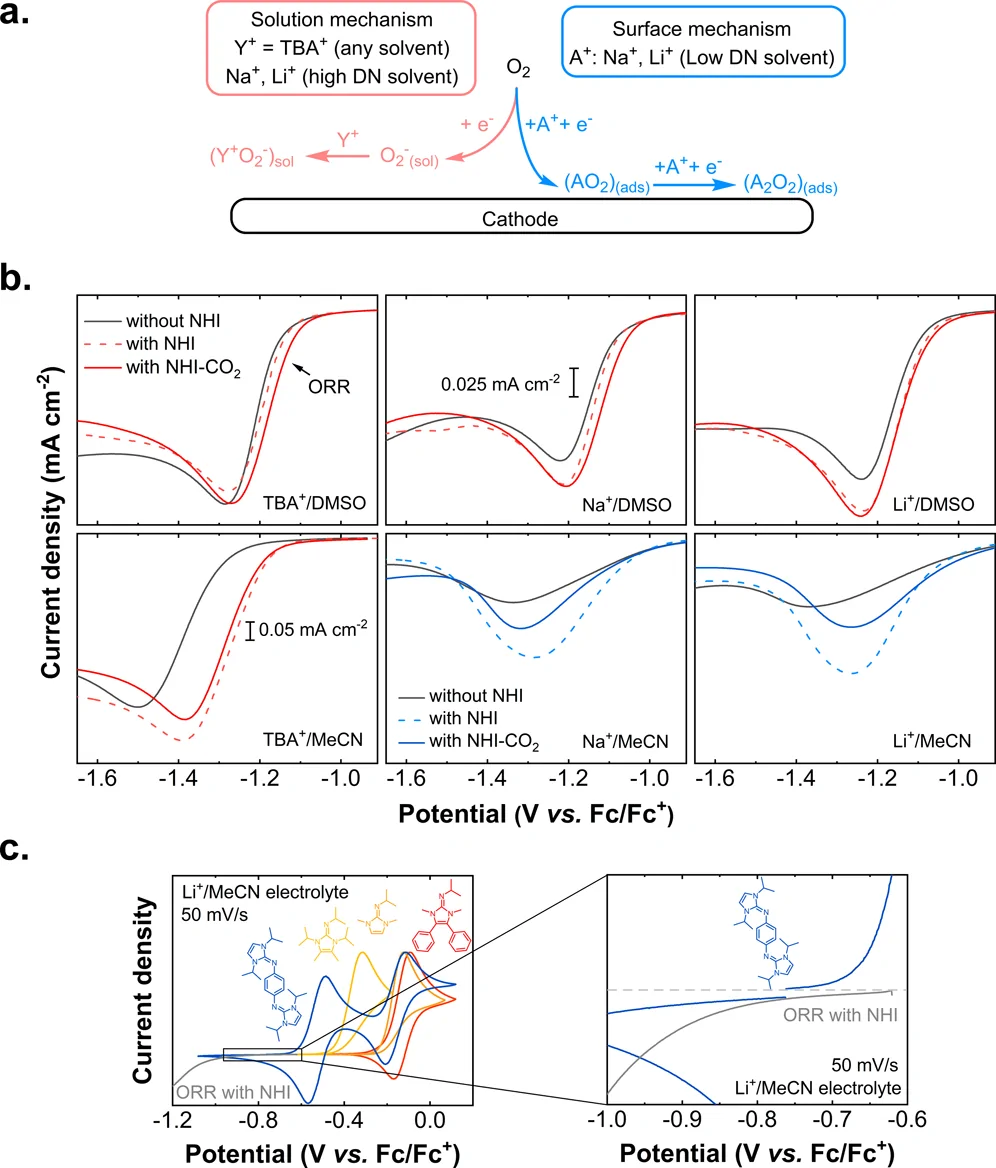
(a) Schematic of solution and surface mechanisms for O_2_ reduction reaction. The predominance of the ORR pathway is highly electrolyte dependent.^35,36^ (b) LSV of the ORR in 0.1 M ATFSI (A = TBA^+^, Na^+^, and Li^+^) in DMSO and MeCN under three conditions: without NHI, with NHI **3**, and with **3-CO**_**2**_ (0.01 M). Working electrode: GC; counter electrode: Pt; reference electrode: Ag/Ag^+^. All LSVs were recorded at a scan rate of 50 mV/s. (c) Overlay of CVs of various 0.01 M NHIs in 0.1 M LiTFSI/MeCN under Ar with LSV of the ORR (17% O_2_) in 0.01 M **3** + 0.1 M LiTFSI/MeCN, which is here taken as representative of typical NHI-shifted ORR potentials. All voltammetry measurements were recorded at a scan rate of 50 mV/s.

LSVs corresponding to ORR in 0.1 M ATFSI (A = TBA^+^, Na^+^, and Li^+^) in DMSO or MeCN were collected under three conditions: O_2_ only; in the presence of 0.01 M NHI **3**; and finally in the presence of 0.01 M **3-CO**_**2**_. We begin with the DMSO systems. As shown in Figure 6b (top row), the reduction peak potentials (∼−1.25 V vs Fc/Fc^+^) of the ORR remain largely unchanged upon the introduction of NHI or NHI–CO_2_ for all three cations (TBA^+^, Li^+^, and Na^+^). As for the peak current, although no obvious change is observed in the TBA^+^/DMSO electrolyte, we note a slight increase (∼15–20%) in peak current in the Na^+^/DMSO and Li^+^/DMSO systems with NHI or NHI–CO_2_. This can be attributed to the strong electron-donating nature of NHI­(−CO_2_), which facilitates the solvation of NaO_2_ or LiO_2_ by interacting with the cation.^42^ This in turn weakens the cation–superoxide interaction and promotes the solution-mediated pathway. However, as the cations are already strongly solvated by DMSO, the observed differences in peak potential and current are relatively small.

In TBA^+^/MeCN electrolyte, the addition of NHI or NHI–CO_2_ again results in a slight increase (8–20%) in peak current; however, this time there is a significant ∼100 mV positive shift in the ORR peak potential due to weak yet significant interactions between TBA^+^ and O_2_^–^ in low donor-number solvents.^41,43^ Furthermore, in Na^+^/MeCN and Li^+^/MeCN electrolytes, the addition of NHI or NHI–CO_2_ produces a slightly larger positive shift in the ORR peak potential (∼100–150 mV), accompanied by a substantially greater increase in peak current (30–100%). We attribute this to the fact that, compared with DMSO where the cations are strongly solvated, interactions between NHI­(−CO_2_) and cations become more pronounced in MeCN due to its weak solvation. Therefore, the additional interaction between NHI­(−CO_2_) and the cation plays a more significant role in weakening cation–superoxide interactions and more effectively promotes the solution-mediated pathway. Note that the increase of the ORR peak current upon NHI­(−CO_2_) addition is not due to an increase in the number of electrons transferred per O_2_ molecule, which remains as 2 e^–^/O_2_ regardless of pathway, but rather because of decreased electrode passivation, as promoting soluble superoxide formation suppresses the accumulation of surface-bound oxide species and preserves a larger electrode active area for ORR.^42,44^ A restatement of this is that the inclusion of NHI or NHI–CO_2_ has a more pronounced effect on the ORR potential and current when the reaction proceeds via a surface-mediated mechanism.

The above findings make clear that having NHI­(−CO_2_) present in solution can unexpectedly modulate the ORR potential to be closer to that of the sorbent. Therefore, comparing NHI–CO_2_ redox with reference ORR data obtained in non-NHI-containing media may miss potential overlap between NHI and shifted O_2_ redox. To evaluate the possibility of unintentional redox overlap upon electrolyte (solvent and salt) variation, CVs of various NHIs in 0.1 M LiTFSI/MeCN electrolyte, with reduction peak potentials ranging from −0.17 to −0.57 V vs Fc/Fc^+^ (representing the range from the highest to lowest potentials of CO_2_-binding NHI examined to date),^12^ are overlaid with a representative LSV of ORR in LiTFSI/MeCN + NHI **3** (Figure 6c) to exemplify a typical degree of NHI-shifted ORR. It is observed that, with electrolyte tuning, the onset potential of ORR begins to approach the oxidation onset potential of certain NHI with particularly negative redox potentials (see the zoomed-in region in Figure 6c). This phenomenon may pose a potential issue, as a further positive shift in ORR could cause oxidation of the NHI by oxygen. To explore how different solvent influences this separation, we extended the above comparison to a high DN solvent system using DMSO (Figure S8). As shown in Figure 6b, the ORR potential is comparatively insensitive to both the addition of the NHI and the cation identity in high DN environments like DMSO. Therefore, a larger potential separation between ORR and NHI oxidation is maintained, making high-DN solvents potentially more favorable for mitigating parasitic oxygen reduction in practical electrolyte design. Therefore, in future studies, it is essential to independently determine the ORR onset potential in the relevant electrolyte system to demonstrate that sufficient separation between NHI and O_2_ redox meets a reasonable operational safety margin.

## Conclusions

This work elucidates the strong role of individual electrolyte components in modulating the thermodynamics of NHI as an EMCC sorbent. We demonstrate that the cation—Na^+^, Li^+^, or Ca^2+^—provides direct stabilization of the NHI–CO_2_ adduct through charge interactions, while the solvent’s donor number (DN) and the anion identity collectively tune the interaction strength. We also found that increasing the magnitude of Δ*H*_NHI–CO2_ can significantly enhance CO_2_ loading, especially at low CO_2_ partial pressure (1000 ppm), which is beneficial for processes operating under an extremely dilute CO_2_ feed stream. Furthermore, we identify a trade-off between capture thermodynamics and electrochemical behavior: strong NHI–CO_2_ and cation interactions effectively stabilize the adduct, although if one is not careful, a kinetic barrier may be imposed that slows electron transfer, hindering the electro-regeneration process. Finally, we highlight that the ORR is strongly modulated not only by the cation and solvent choices but also by NHI, which can promote a solution-mediated pathway and shifts the ORR onset potential positively. Further positive shifts may enable O_2_ to oxidize NHIs, particularly those with the most negative reduction potentials. This places the burden on researchers to ensure that stability is demonstrated in each new investigated electrolyte system rather than assuming a universal redox potential applies, which is too simplistic. Overall, future electrolyte engineering must adopt a holistic perspective, balancing capture thermodynamics with electrochemical redox behaviors to optimize integrated carbon management systems.

## Supplementary Material



## Notes

Experimental methods, Raman spectra of 0.1 M LiOTf in the absence and presence of 0.1 M NHI–CO_2_, chart of solvent donor numbers and dielectric constants, schematic of the experimental setup for measuring CO_2_ loading of NHI, and chemical structures of NHIs used for CV analysis (PDF)
