# Supplementary material for: Electrolyte Effects on N‑Heterocyclic Imines as the Redox Sorbent for Electrochemical CO2 Capture
Source: ACS Cent Sci. 2026 Sep 3;12(9):1410–7. doi: 10.1021/acscentsci.6c00765 (PMC13614030; doi:10.1021/acscentsci.6c00765)
Supplement: Supplementary file 1 [file oc6c00765_si_001.pdf]

Supporting Information

Electrolyte Effects on N-heterocyclic Imines as the Redox Sorbent for Electrochemical CO_2 Capture

Fang-Yu Kuo[a] , *Gi Hyun Byun*[b], *Betar M. Gallant*[b*]

[a] Department of Chemical Engineering, Massachusetts Institute of Technology, Cambridge, Massachusetts 02139, United States

[b] Department of Mechanical Engineering, Massachusetts Institute of Technology, Cambridge, Massachusetts 02139, United States

[*] Corresponding author

EXPERIMENTAL METHODS

Safety Statement: no unexpected or unusually high safety hazards were encountered.

Materials Preparation. N-heterocyclic imines used in this study were synthesized according to synthesis procedures reported in our previous study.[1] TBATFSI (>99.0%, Sigma-Aldrich), $TBAPF_6$ (>99.0%, Sigma-Aldrich), TBAOTf (>99.0%, Sigma-Aldrich), NaTFSI (99.5%, Solvionic), LiTFSI (99.99%, Sigam-Aldrich), $LiPF_6$ (≥99.99%, Sigma-Aldrich), $LiBF_4$ (99.99%, Sigma-Aldrich), $LiClO_4$ (99.99%, Sigam-Aldrich), LiOTf (99.995%, Sigma-Aldrich), and $Ca(TFSI)_2$ (99.5%, Solvionic) were dried in a Buchi glass oven under active vacuum at 120 °C for 24 h before transfer to the glovebox (H_2O content <0.1 ppm, O_2 content <0.1 ppm, MBRAUN) and used without further purification. 3Å molecular sieves (bead size 8-12 mesh, Sigma-Aldrich) were activated in the Buchi glass oven under active vacuum for 24 hours at 250 °C. All solvents (acetonitrile (MeCN), propylene carbonate (PC), γ-butyrolactone (GBL), dimethylformamide (DMF), and dimethyl sulfoxide (DMSO)) were all purchased from Sigma-Aldrich and dried over 3Å molecular sieves over 48 h to remove the residual water; water content was determined to be <10 ppm by KF titrator before preparing the electrolyte. All sample preparation and storage were conducted inside the glovebox.

Reaction Microcalorimetry. Reaction enthalpies were measured using a Micro Reaction Calorimeter (uRC™, Thermal Hazard Technology) configured with a continuous gas-flow module. For each measurement, 0.7 mL of 0.1 M NHI in electrolyte with desired composition

was introduced into a 1.5 ml stainless-steel vial. A stream of pure CO_2 (1 sccm) was then passed through the solution at 1 atm until the thermal signal stabilized, signifying completion of CO_2 uptake. The use of pure CO_2 is to ensure full CO_2 loading of NHI, enabling straightforward enthalpy normalization and comparison. The reaction enthalpy was obtained by integrating the recorded heat flow over time. To isolate the heat associated with NHI-CO_2 binding, all values were corrected by subtracting the CO_2 dissolution enthalpy of the corresponding neat solvent, measured separately under identical conditions.

Nuclear Magnetic Resonance (NMR) Spectroscopy. The NMR samples are prepared and loaded in a capped Wilmad NMR tube (600 μL) and Bruker Avance Neo 500 MHz was used to collect the 1H, 7Li, and 13C NMR spectra. Diffusion parameters (D20 and P30) in the diffusion-order spectroscopy (DOSY) NMR pulse sequence are tuned for each analyte to provide 90 – 95 % signal attenuation.

Raman Spectroscopy. Electrolyte solutions were prepared and sealed in glass tubes under an argon atmosphere inside a glovebox to prevent air exposure. Raman measurements were conducted directly on the sealed samples using a Renishaw Invia Reflex confocal Raman microscope. The laser source used was a 532 nm laser operated at 50 mW, coupled with a 1200 lines/mm diffraction grating. Multiple spectral acquisitions were conducted and accumulated to enhance signal quality. All spectra were normalized against the MeCN C–C stretching band at 918 cm^{-1}.

CO_2 Loading Measurements. Sorbent solutions/electrolytes (3 mL) of the desired composition were loaded into gas-tight headspace vials (SureSTART™ 10 mL glass screw-top) in the Ar glovebox. During the measurement, the vial septum was pierced with an inlet needle connected to mass flow controllers that delivered a total gas flow of 10 sccm at 1 atm, with the CO_2/N_2 ratio adjusted to set the inlet CO_2 concentration. A second needle provided the outlet stream to an infrared CO_2 sensor (CO2Meter), which was calibrated prior to each experiment. Gas was continuously passed through the solution until the outlet CO_2 signal returned to its baseline level, indicating saturation. The total CO_2 uptake was determined by integrating the concentration–time profile.

Calculation of the Equilibrium Constant K and Gibbs Free Energy. The equilibrium constant for CO_2 binding (K) was defined based on the following chemical equilibrium,

$$NHI + CO_2 \overset{K}{\leftrightarrow} NHICO_2 \tag{1}$$

$$K = \frac{[NHICO_2]}{[NHI]p_{CO2}} \tag{2}$$

where [$NHICO_2$] is the concentration of the CO_2 loaded NHI, [NHI] is the concentration of lean NHI, and pCO_2 is the partial pressure of the CO_2 of the experiment. The experimentally measured CO_2 loading, denoted as θ (ranging from 0 to 1), represents the fraction of CO_2-bound adduct, such that $[NHICO_2]/[NHI]_0 = \theta$ and $[NHI]/[NHI]_0 = 1-\theta$, where $[NHI]_0$ is the initial or total concentration of all NHI species. Therefore, the equilibrium constant can be written as

$$K = \frac{\theta}{(1-\theta)p_{CO2}} \quad (3)$$

With further rearrangement, we can obtain a Langmuir-like expression

$$\theta = \frac{K \cdot p_{CO2}}{1+K \cdot p_{CO2}} \quad (4)$$

A single fitted value of K was obtained by fitting the CO_2 loading data (θ) across various CO_2 partial pressure to the above equation (4). Because the experimentally measured uncertainty among various measured p_{CO2} range is different, a nonlinear weighted least squares (WLS) regression was employed to handle the influence of data points with different variances. The regression was performed using the "nlinfit" function in MATLAB. The objective function minimized the weighted sum of squared residuals, utilizing statistical weights defined as $W_i = 1/\sigma_i^2$, where σ_i is the experimental standard deviation of the measured CO_2 loading at each corresponding pressure.

The 95% confidence interval (CI) for the fitted parameter K was evaluated using MATLAB's "nlparci" function, which derives the symmetric parameter uncertainty based on the Jacobian matrix and residuals from the fit. The corresponding standard Gibbs free energy change was calculated using the following

$$\Delta G = -RT ln\, K \quad (5)$$

where R is the ideal gas constant and T is the experimental temperature. We would like to note that the uncertainty of K is symmetric, however, propagating this error through natural

logarithm for calculation of ΔG will result in asymmetric uncertainty. To report a single, conservative error margin (±) for ΔG, the larger absolute difference between the best-fit value and the upper/lower bounds was selected.

Electrochemical Characterization. Cyclic voltammetry and linear sweep voltammetry (LSV) were performed in a low-volume glass three-electrode cell (Pine Research) equipped with a glassy carbon working electrode, a platinum counter electrode, and an Ag/Ag^{+} pseudo-reference electrode (Pine Research, LowProfile glass frit). For NHI measurements, electrolytes were prepared inside an argon glovebox for Ar-condition experiments and subsequently saturated with pure CO_2 for CO_2-condition measurements. For oxygen reduction studies, the electrolyte was sparged for 10 minutes prior to LSV measurements using a 4:1 mixture of compressed air with N_2 (for samples without NHI and with NHI) or CO_2 (for samples containing NHI–CO_2). All reported potentials were referenced to Fc/Fc^{+} using ferrocene as an external calibration standard. All measurements are conducted with 85% IR correction.

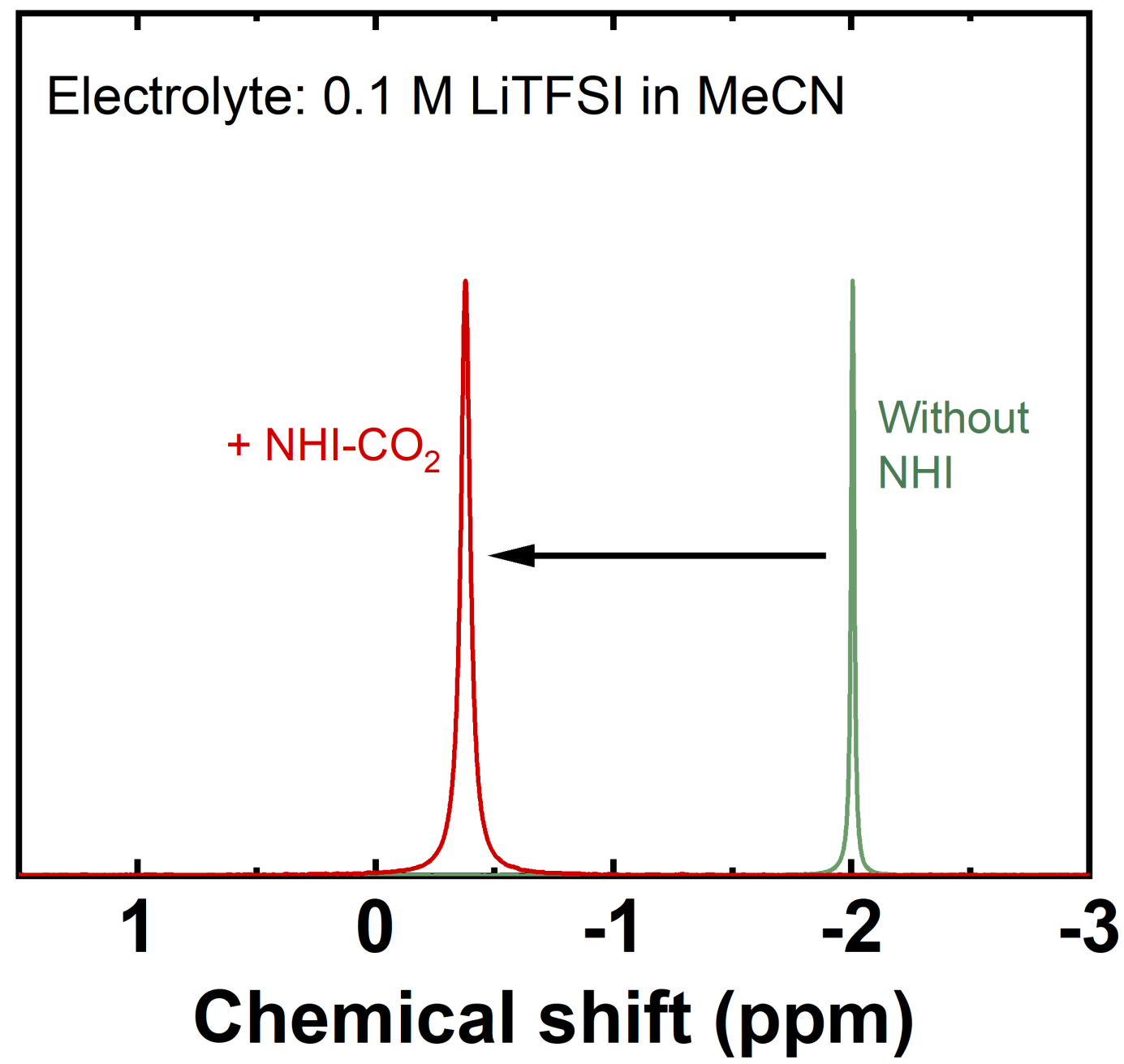

Figure S1. 7Li NMR of 0.1 M LiTFSI in MeCN and 0.1 M NHI-CO_2 in 0.1 M LiTFSI/MeCN.

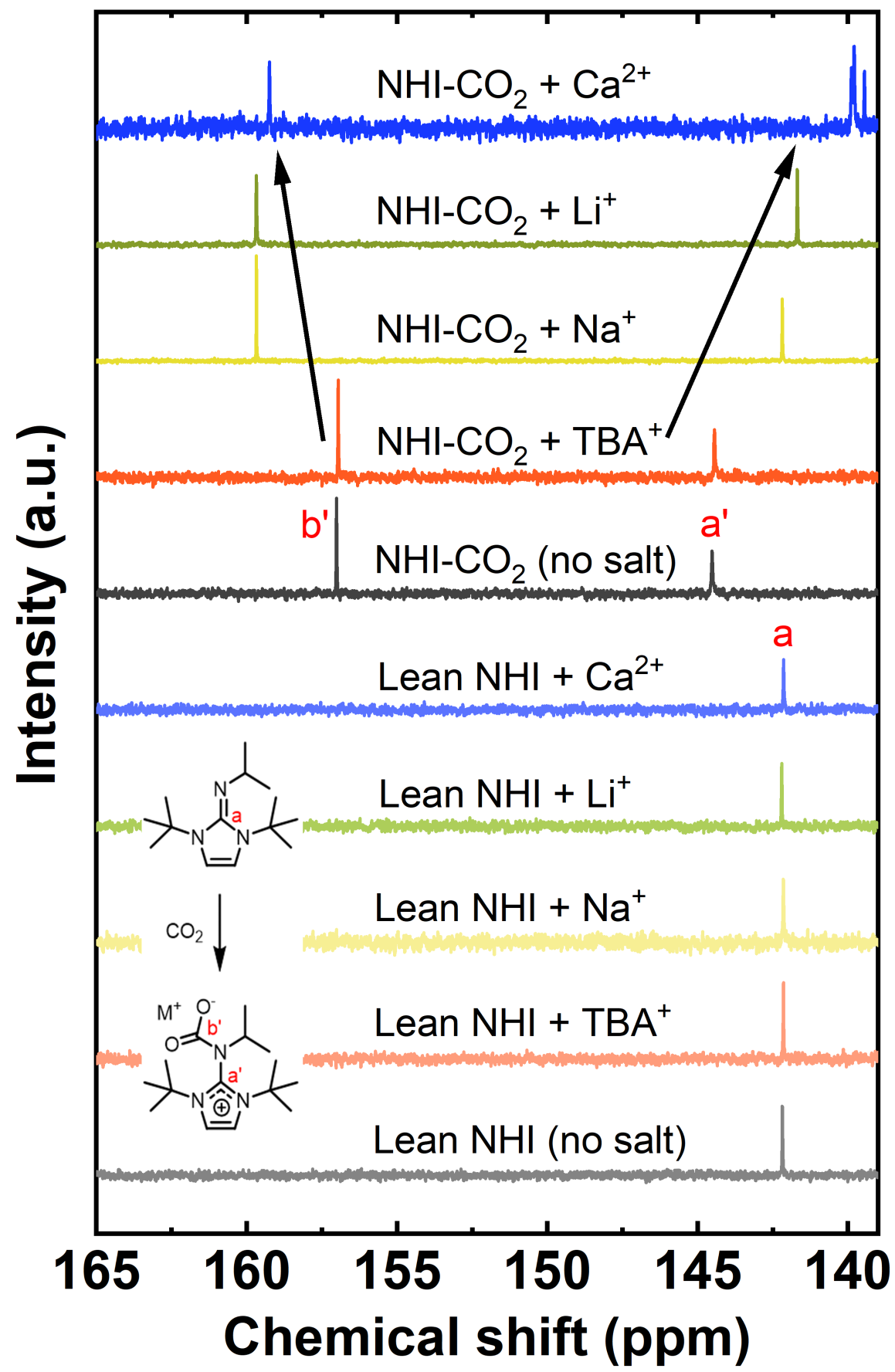

Figure S2. ^{13}C NMR spectra of lean NHI and CO_2-loaded NHI (0.1 M) in salt-free MeCN and in the presence of 0.1 M TBA^+, Na^+, Li^+, and Ca^{2+} salts.

Discussion:

Figure S2 shows the ^{13}C NMR spectra of lean and CO_2-loaded NHI in MeCN with various electrolyte cations. Upon CO_2 loading, the **b′** resonance shifts downfield relative to the salt-free and TBA^+ cases, consistent with cation interaction with the carbamate moiety. However, this downfield shift reaches a maximum with Na^+ and does not increase further with Li^+ or Ca^{2+}, indicating that **b′** is not a strictly monotonic reporter of cation Lewis acidity beyond Na^+. In contrast, the **a′** resonance exhibits a monotonic upfield shift as the cation Lewis acidity increases from TBA^+ to Na^+, Li^+, and Ca^{2+}. This trend is attributed to stronger cation coordination to the carbamate oxygen atoms, which stabilizes the anionic carbamate moiety and reduces charge separation within the zwitterionic NHI-CO_2 adduct. The resulting charge redistribution increases electron density at the NHI core, leading to the observed upfield shift of **a′** and supporting stronger overall adduct stabilization. In the Ca^{2+} case, the **a′**

resonance broadens and splits into multiple peaks, likely reflecting multiple Ca^{2+}-associated adduct environments with different coordination modes or stoichiometries.

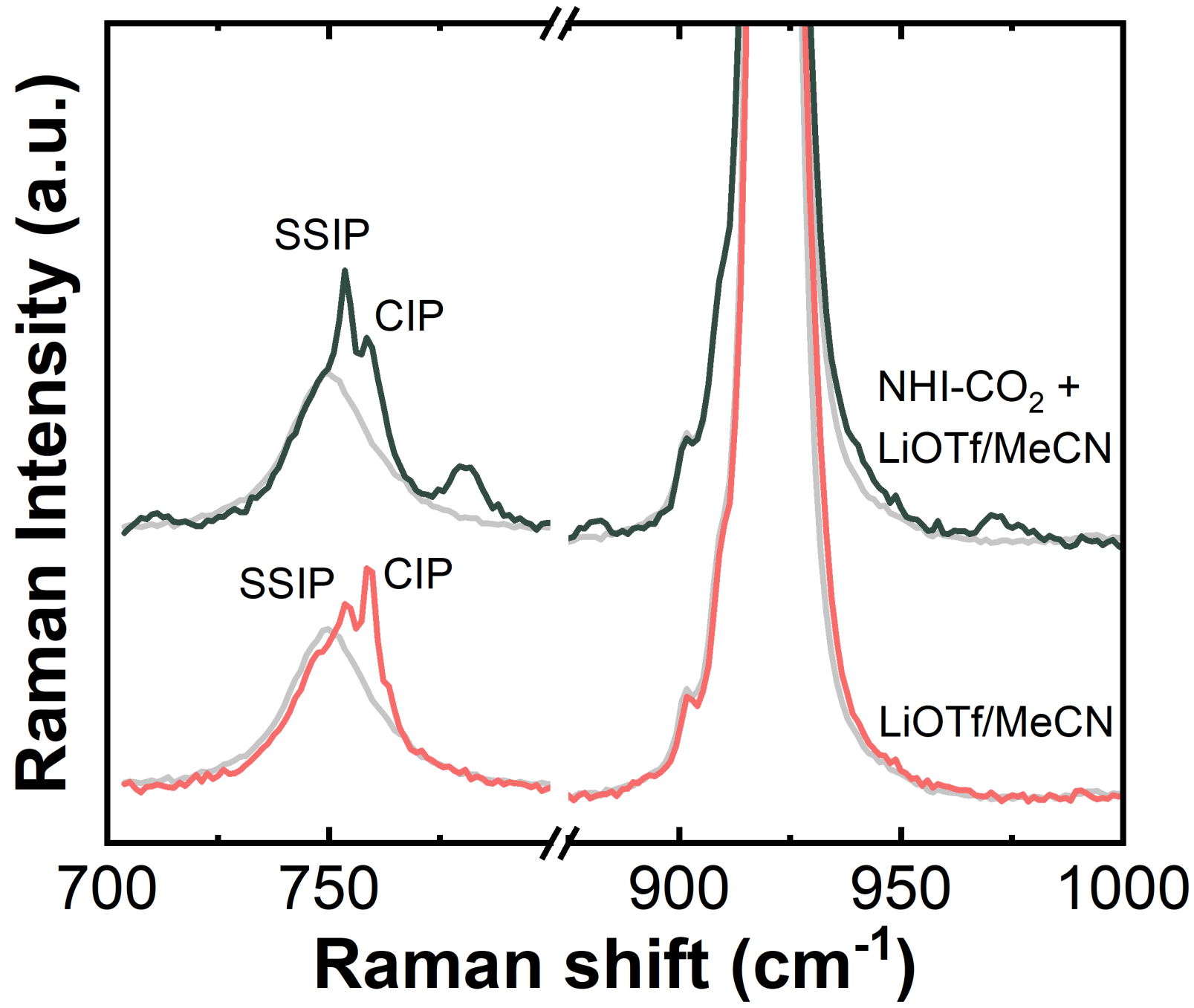

Figure S3. Raman spectra of 0.1 M LiOTF/MeCN electrolyte with and without 0.1 M NHI-CO_2.

Discussion:

As shown in Figure S3, the addition of 0.1 M NHI-CO_2 into 0.1 M LiOTf/MeCN electrolyte leads to the increase of the SSIP and decrease of the CIP population. As the Raman shift near 760 cm^{-1} corresponding to the solvation state of the OTf^- anion, this result indicates that NHI-CO_2 replaces OTf^- in the first solvation shell of Li^+, leading to the increase of the solvent-separated OTf^- anion. This spontaneous exchange reaction also indicates that the carbamate anion on the NHI-CO_2 adduct is a stronger electron donor compared to the OTf^- anion, and the exchange leads to a more stabilized Li^+ solvation environment and release additional heat in the reaction enthalpy measurement.

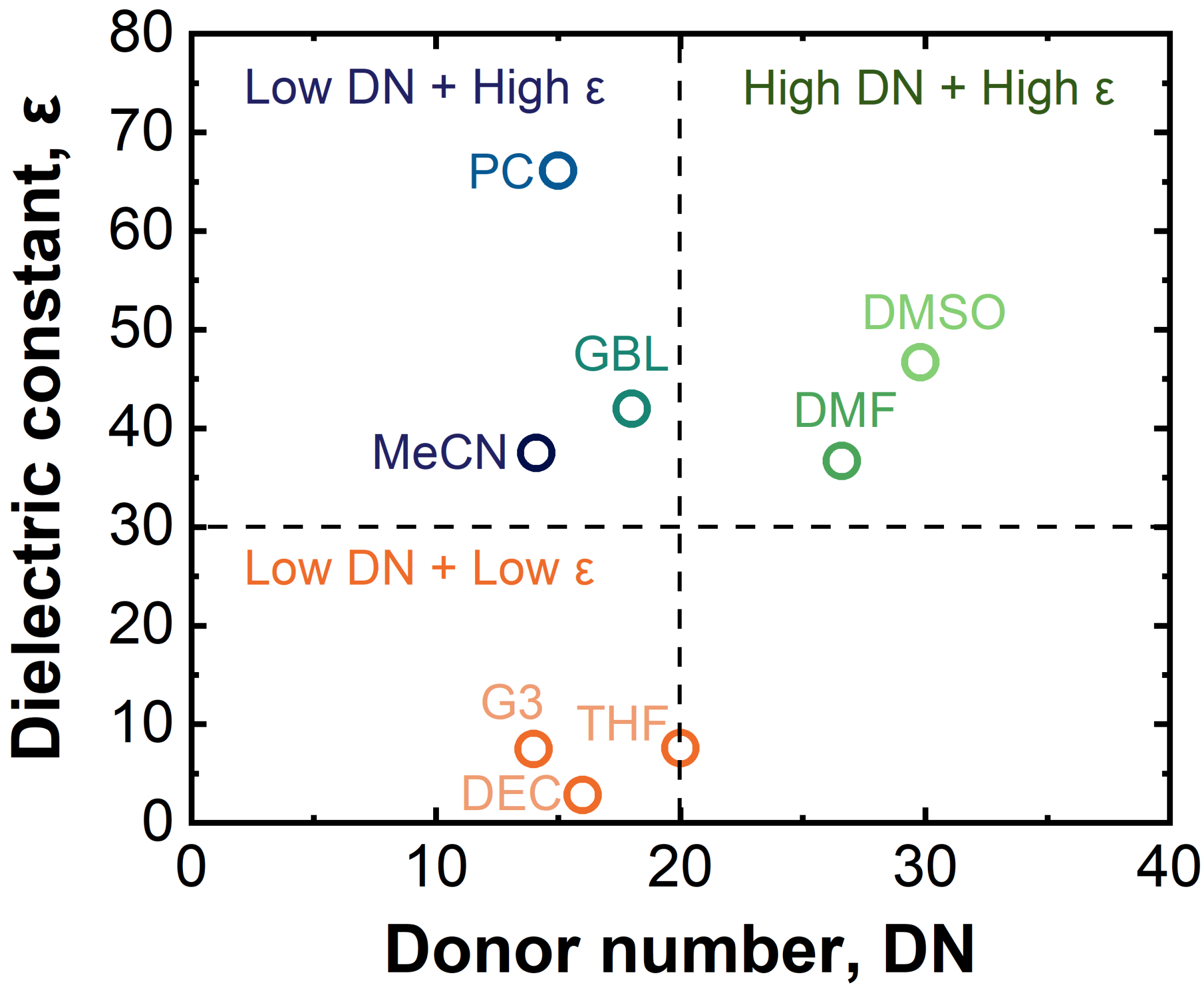

Figure S4. Solvent donor number and dielectric constants.

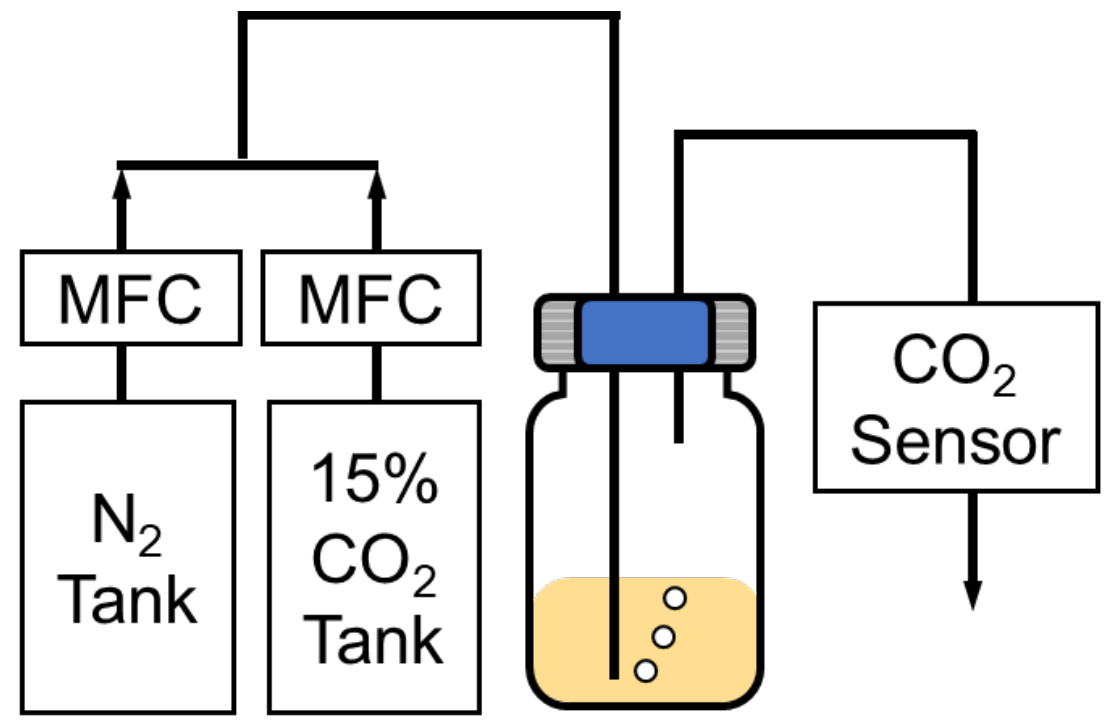

Figure S5. Schematic illustration of experimental setup for measuring the CO_2 loading of the NHI.

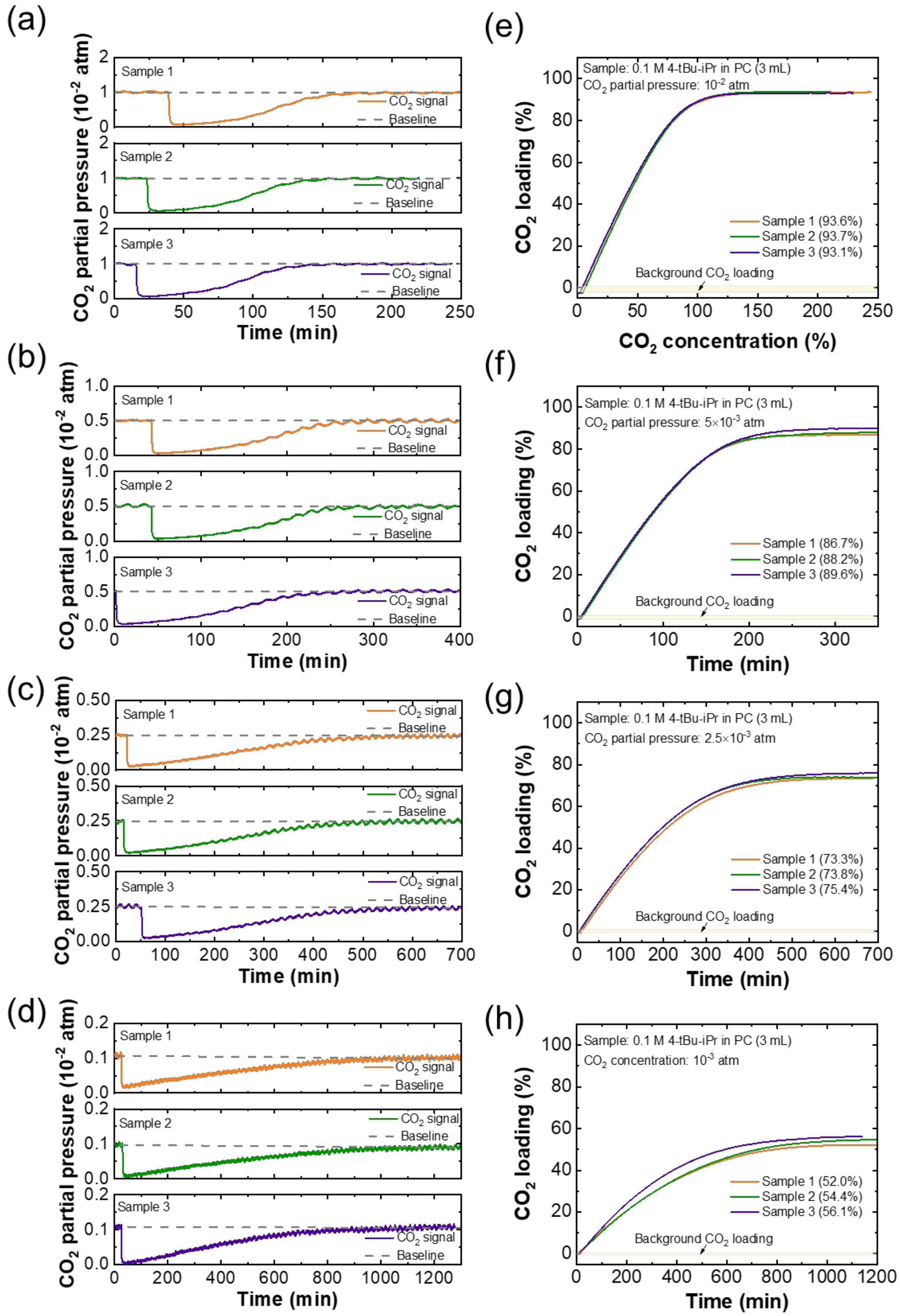

Figure S6. CO_2 partial pressure changes during capture by 0.1 M 4-tBu-iPr in PC (3 mL) under feed streams containing (a) 10^{-2}, (b) 5×10^{-3}, (c) 2.5×10^{-3}, and (d) 10^{-3} atm CO_2 partial pressure. Corresponding CO_2 loading profiles are shown for feed streams containing (e) 10^{-2}, (f) 5×10^{-3}, (g) 2.5×10^{-3}, and (h) 10^{-3} atm CO_2 partial pressure.

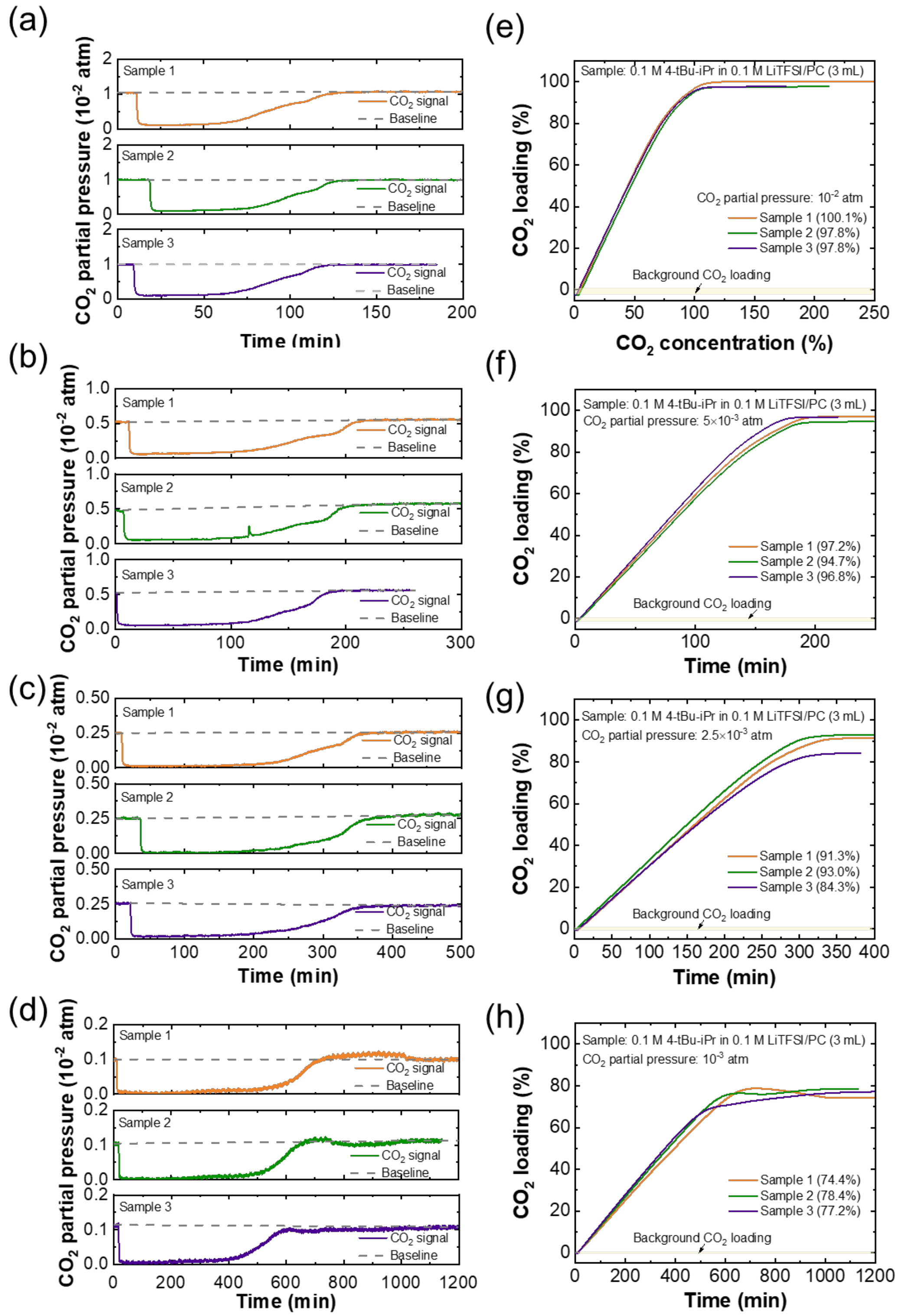

Figure S7. CO_2 partial pressure changes during capture by 0.1 M 4-tBu-iPr in 0.1 M LiTFSI/PC (3 mL) under feed streams containing (a) 10^{-2}, (b) 5×10^{-3}, (c) 2.5×10^{-3}, and (d) 10^{-3} atm CO_2 partial pressure. Corresponding CO_2 loading profiles are shown for feed streams containing (e) 10^{-2}, (f) 5×10^{-3}, (g) 2.5×10^{-3}, and (h) 10^{-3} atm CO_2 partial pressure.

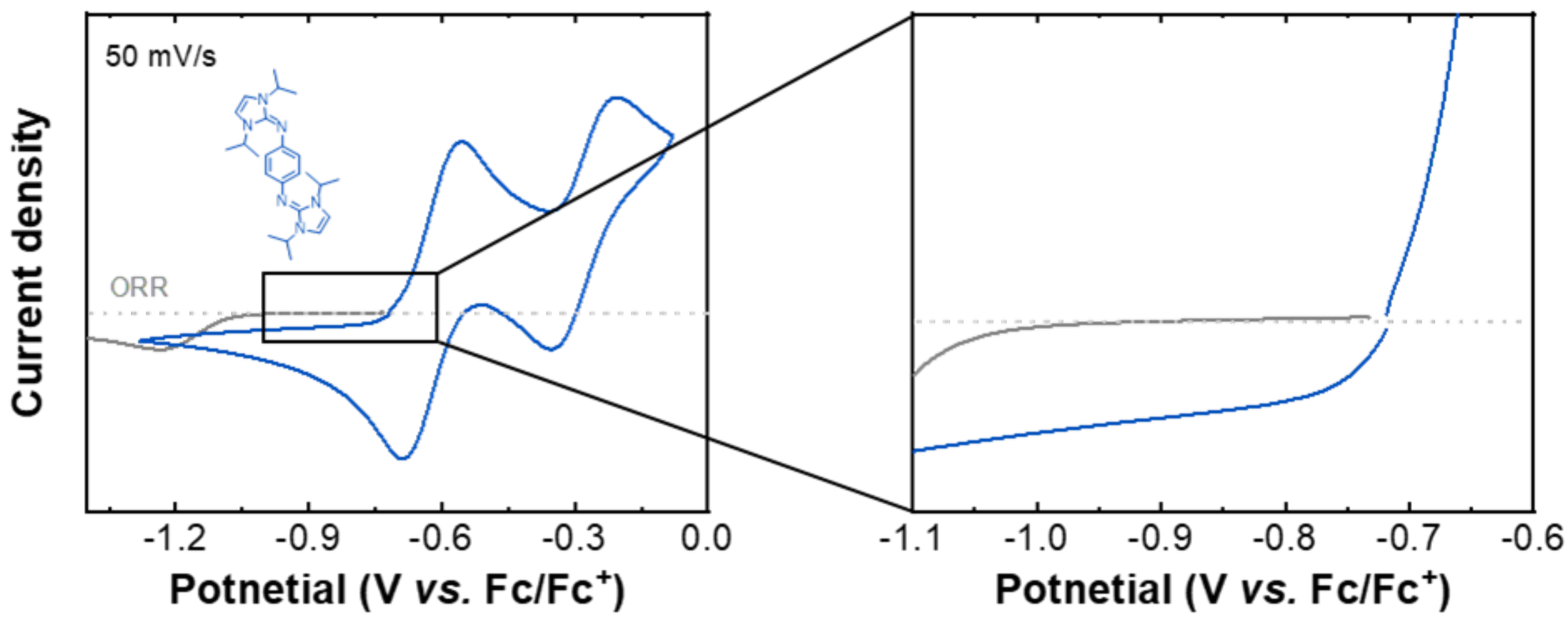

Figure S8. Overlay of CVs of NHI in 0.1 M LiTFSI/DMSO under Ar with LSV of the ORR (17% O_2) in 0.01 M NHI **3** + 0.1 M LiTFSI/DMSO. All voltammetry measurements were recorded at a scan rate of 50 mV/s.

Reference

(1) Kuo, F.-Y.; Byun, G. H.; Obi, A. D.; Junor, G. P.; Gallant, B. M. Oxygen-tolerant electrochemical CO2 separation using N-heterocyclic imines with superstoichiometric release per electron. Nature Energy 2026, 1-9.
